# Supplementary material for: Global biogeographic regions for ants have complex relationships with those for plants and tetrapods
Source: Nat Commun. 2024 Jul 5;15:5641. doi: 10.1038/s41467-024-49918-2 (PMC11226674; doi:10.1038/s41467-024-49918-2)
Supplement: Supplementary file 1 — Supplementary Information [file 41467_2024_49918_MOESM1_ESM.pdf]

SUPPLEMENTARY

Global biogeographic regions for ants have complex relationships with those for plants and tetrapods

Runxi Wang, Jamie M. Kass, Chhaya Chaudhary, Evan P. Economo and Benoit Guénard

Corresponding should be addressed to R. W. (email: runxiwg@connect.hku.hk)

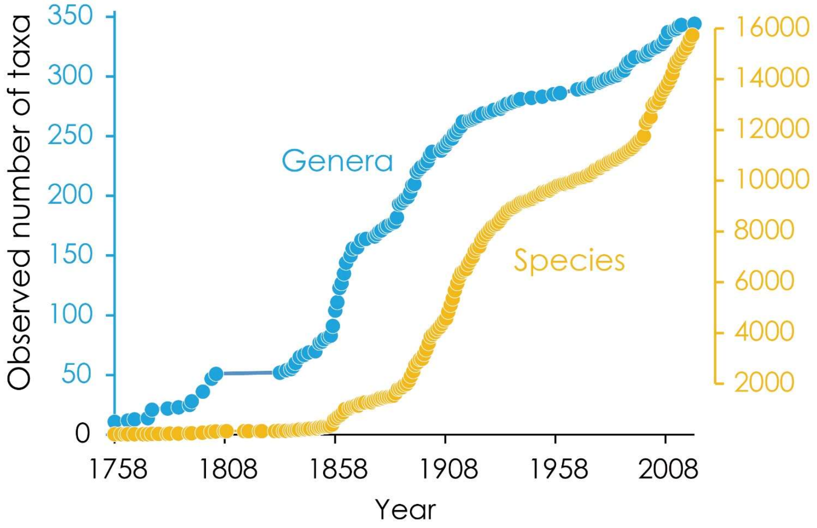

Supplementary Fig.1 Taxonomic accumulation of ant genus and species-level descriptions over time (1758 – 2021) for valid taxa. The observed number of taxa (genera and species) is used to indicate the taxonomic activity levels. Time of the specific taxon established is used, only valid taxa are included. Data is from the Global Ant Biodiversity Informatics (GABI, Guénard et al., 2017) with taxonomy following Bolton (2022).

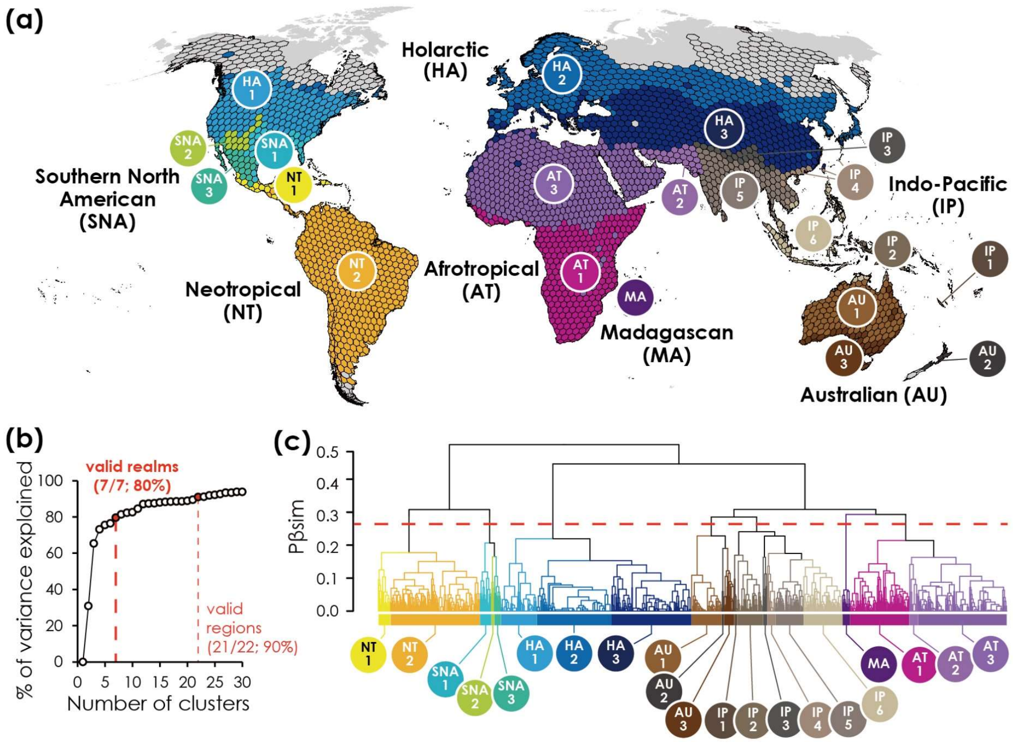

Supplementary Fig.2 Delineation and clustering results of ant assemblages based on generic phylogenetic turnover (Pβsim). Map of ant biogeographic units delineated (a), percentage of variance explained by the number of clusters (b) and dendrogram (c) resulting from the unweighted pair-group method using arithmetic average (UPGMA) hierarchical clustering based on different geographic units. Grey color indicates areas without sufficient data or invalid biogeographic units. Map is projected in Robinson projection system.

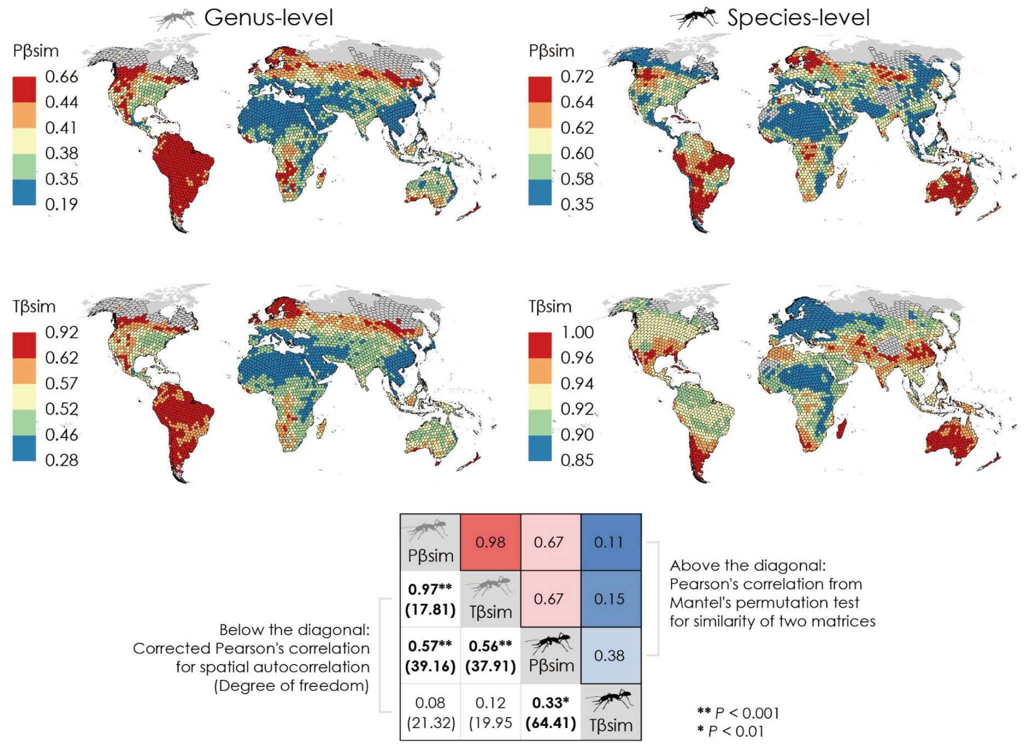

Supplementary Fig.3 Spatial patterns and congruence of phylogenetic and taxonomic turnover (Pβsim and Tβsim). The congruence of Pβsim and Tβsim is tested by mantel's permutation test (999 times) and modified t-test corrected for spatial autocorrelation. The darker red indicates the higher value of correlation coefficient while darker blue indicates the lower value.

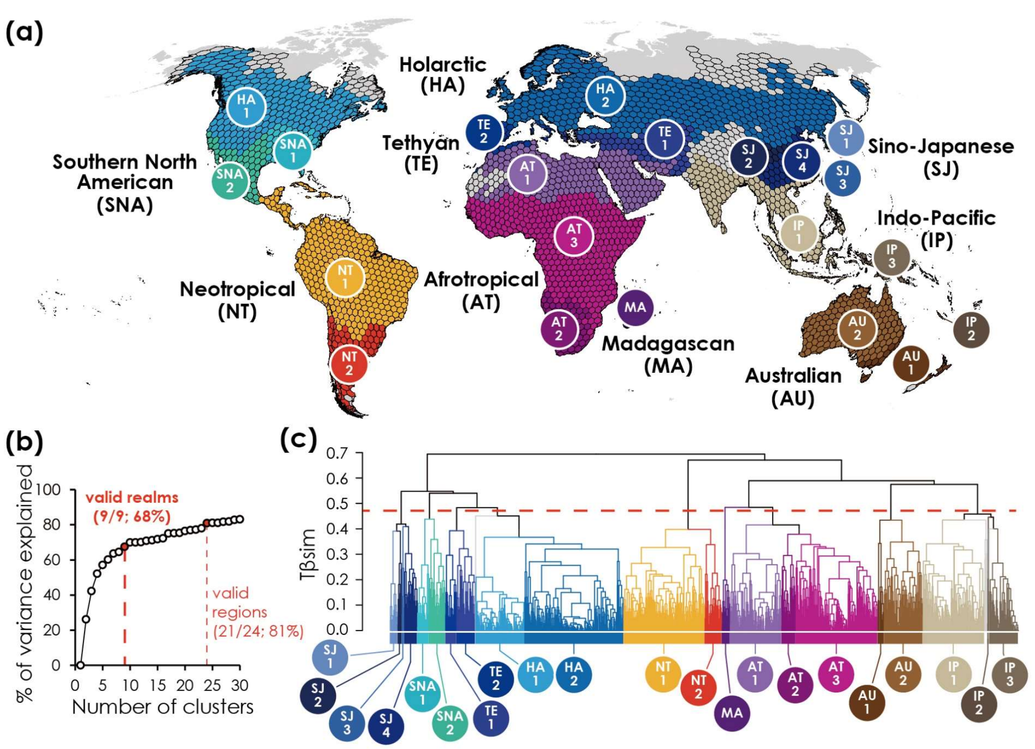

Supplementary Fig.4 Delineation and clustering results of ant assemblages based on species-level phylogenetic turnover (Pβsim). Map of ant biogeographic units delineated (a), percentage of variance explained by the number of clusters (b) and dendrogram (c) resulting from the unweighted pair-group method using arithmetic average (UPGMA) hierarchical clustering based on different geographic units. Grey color indicates areas without sufficient data or invalid biogeographic units. Map is projected in Robinson projection system.

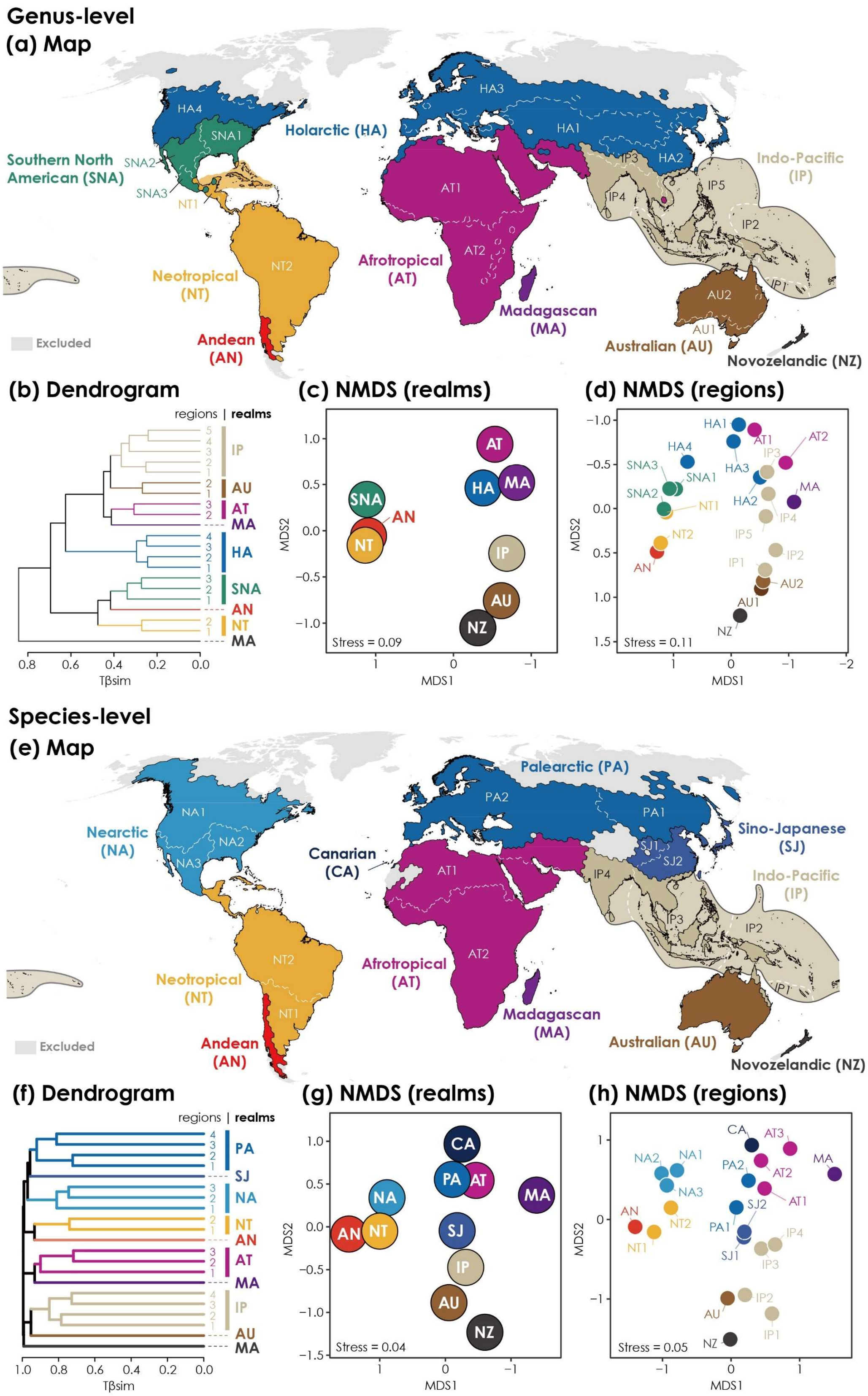

Supplementary Fig.5 Taxonomic biogeographic classifications of ants of the world. Both

regionalization at genus- (a-d) and species-level (e-h) is presented. Map of ant biogeographic realms and regions of the world (a) and their dendrogram (c) resulting from the unweighted pair-group method using arithmetic average (UPGMA) hierarchical clustering based on phylogenetic turnover (Pβsim) across 5×10^4 km^2 hexagons, and the relationships among realms (c) and regions (d) resulting from the nonmetric multidimensional scaling (NMDS) ordination. Black solid lines and white lines (dashed on the ocean) delineate the borders of realms (9 and 11 at genus- and species-level, respectively) and 21 regions. Colors used to characterize particular realms in maps, dendrograms and ordination plots are identical. Grey color indicates areas without sufficient data or invalid biogeographic units. Map is projected in Robinson projection system.

Supplementary Figs. 6-13 Sensitivity of dissimilarity metrics, distributional data and clustering analyses

in regionalization. Decision of number of clusters are based on the 'elbow' method according to the range of explained variance (up to 30 clusters). The number of clusters correspond to the 'elbow' point (i.e., point of optimal curvature) is considered as the number of biogeographic units. If biogeographic units have enough resolution (i.e., regions are recognized), they are further grouped into higher level unit (i.e., realms). However, the large numbers of clusters are not chosen because it would hinder the interpretation of bioregionalization in the large-scale. The obtained biogeographic unit is considered as invalid if it only includes very few numbers of hexagons or not characterized by distinct and coherent geographic units that can be delineated clearly in space.

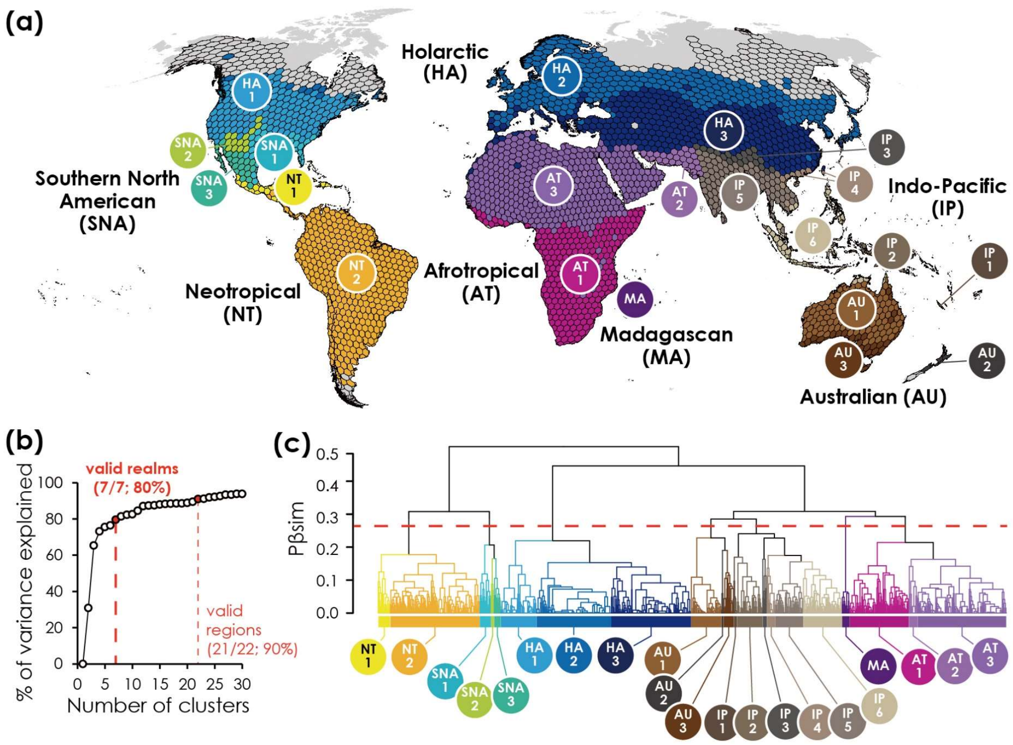

Supplementary Fig.6 Biogeographic regionalization of ant genera based on taxonomic turnover (Tβsim). Map of ant biogeographic units delineated (a), percentage of variance explained by the number of clusters (b) and dendrogram (c) resulting from the unweighted pair-group method using arithmetic average (UPGMA) hierarchical clustering based on different geographic units. Colors used to characterize particular realms in maps and dendrograms are identical. Grey color indicates areas without sufficient data or invalid biogeographic units. Map is projected in Robinson projection system.

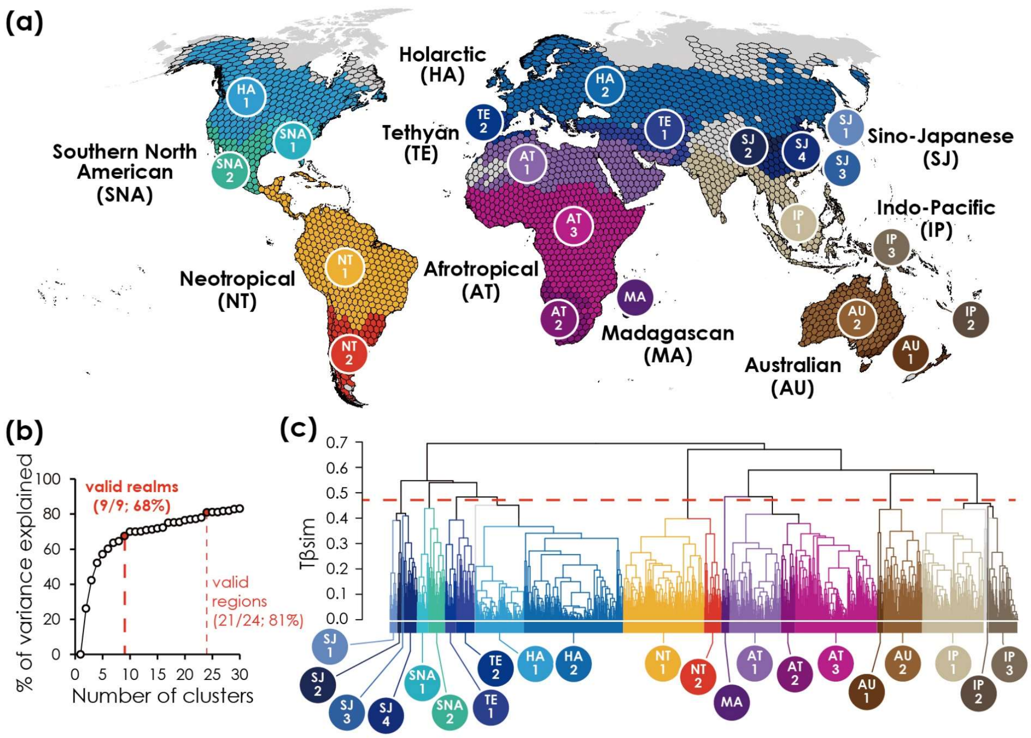

Supplementary Fig.7 Biogeographic regionalization of ant species based on taxonomic turnover (Tβsim). Map of ant biogeographic units delineated (a), percentage of variance explained by the number of clusters (b) and dendrogram (c) resulting from the unweighted pair-group method using arithmetic average (UPGMA) hierarchical clustering based on different geographic units. Colors used to characterize particular realms in maps and dendrograms are identical. Grey color indicates areas without sufficient data or invalid biogeographic units. Map is projected in Robinson projection system.

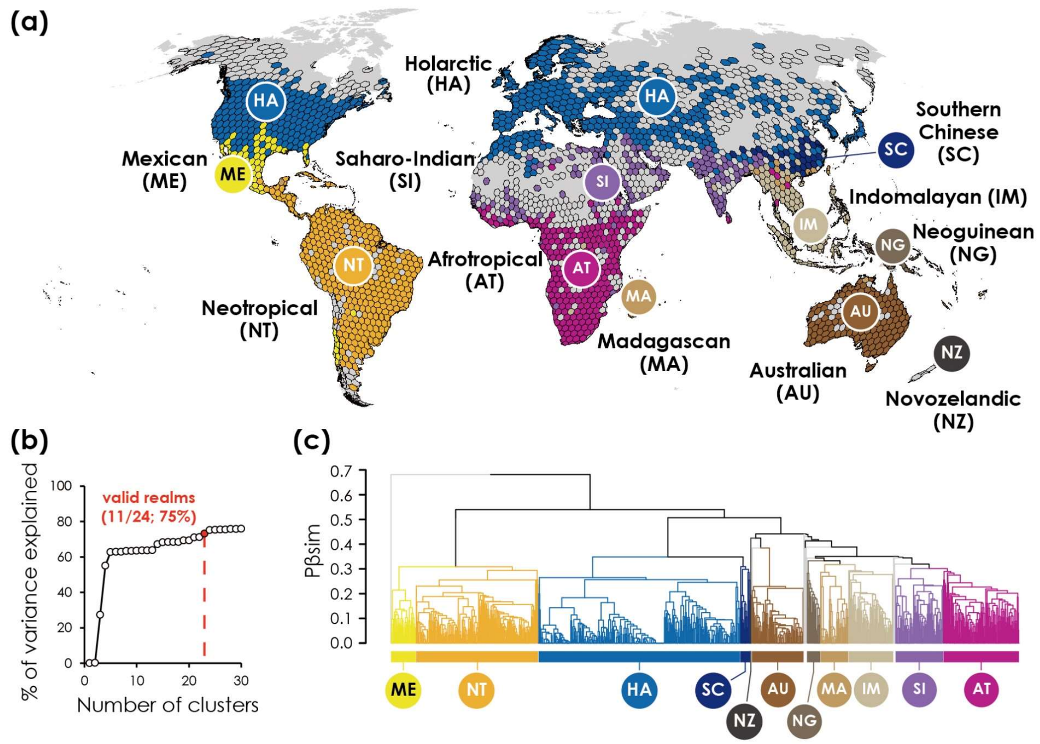

Supplementary Fig.8 Biogeographic regionalization of ant genera based on phylogenetic turnover (Pβsim) and raw distributional records of hexagons. Map of ant biogeographic units delineated (a), percentage of variance explained by the number of clusters (b) and dendrogram (c) resulting from the unweighted pair-group method using arithmetic average (UPGMA) hierarchical clustering based on different geographic units. Colors used to characterize particular realms in maps and dendrograms are identical. Grey color indicates areas without sufficient data or invalid biogeographic units. Map is projected in Robinson projection system.

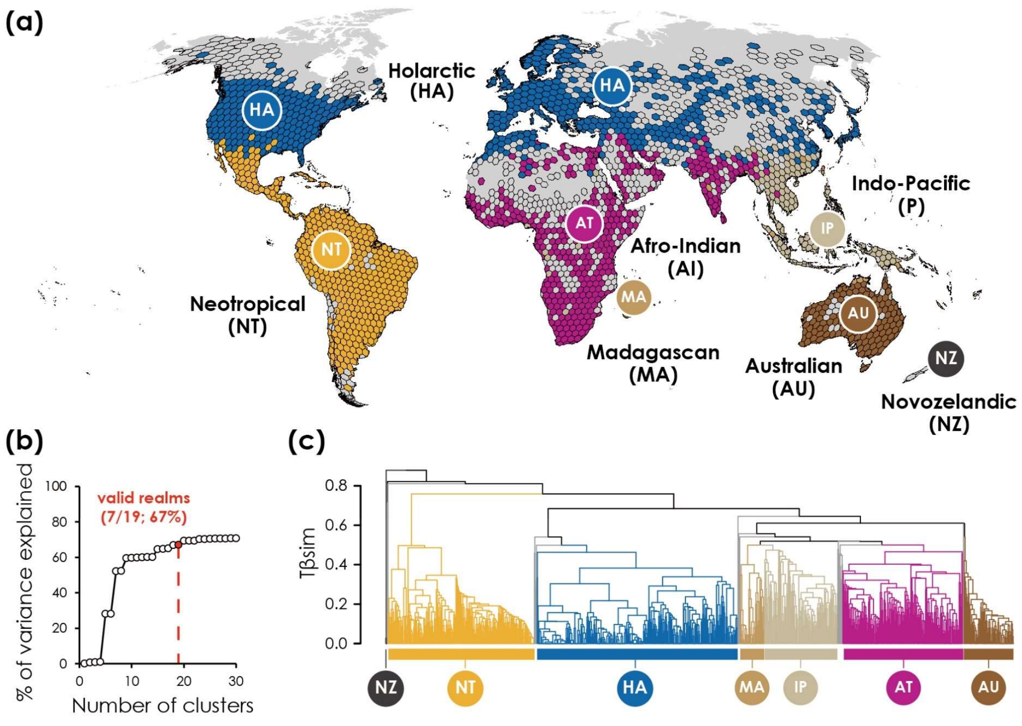

Supplementary Fig.9 Biogeographic regionalization of ant genera based on taxonomic turnover (Tβsim) and raw distributional records of hexagons. Map of ant biogeographic units delineated (a), percentage of variance explained by the number of clusters (b) and dendrogram (c) resulting from the unweighted pair-group method using arithmetic average (UPGMA) hierarchical clustering based on different geographic units. Colors used to characterize particular realms in maps and dendrograms are identical. Grey color indicates areas without sufficient data or invalid biogeographic units. Map is projected in Robinson projection system.

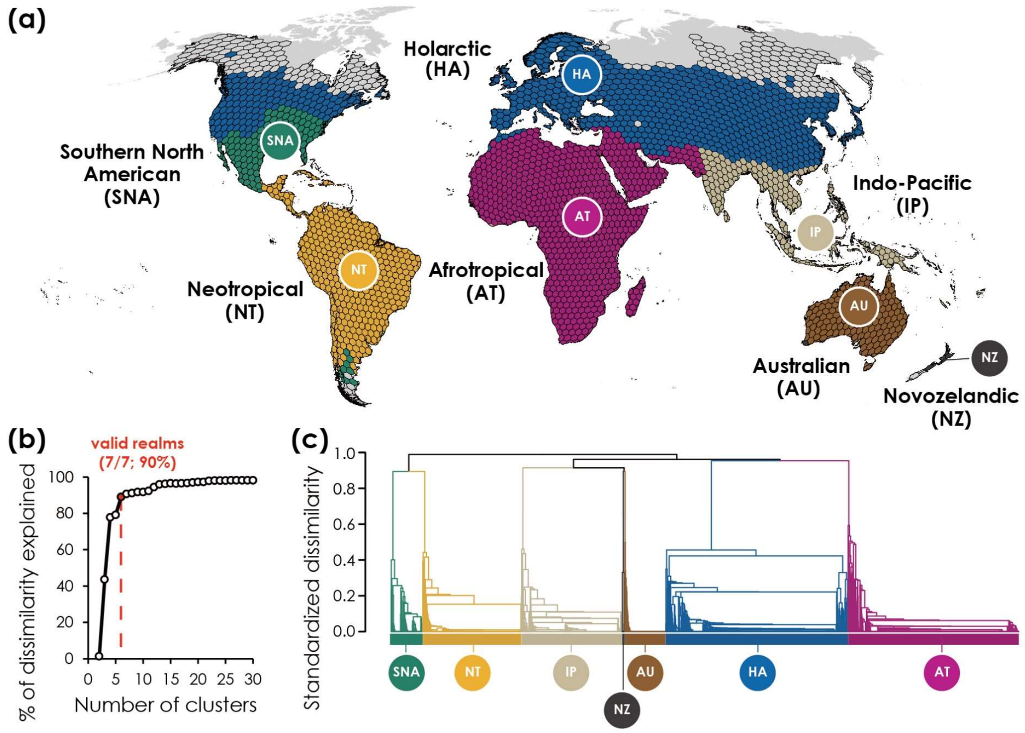

Supplementary Fig.10 Biogeographic regionalization of ant genera based on phylogenetic turnover (Pβsim) by using unbiased clustering algorithm. Map of ant biogeographic units delineated (a), percentage of standardized dissimilarity explained by the number of clusters (b) and dendrogram (c) resulting from the unweighted pair-group method using arithmetic average (UPGMA) hierarchical clustering based on different geographic units. The standardized dissimilarity presented is the frequency of each pair being located in the specific number of clusters over 100 dendrogram trees. Colors used to characterize particular realms in maps and dendrograms are identical. Grey color indicates areas without sufficient data or invalid biogeographic units. Map is projected in Robinson projection system.

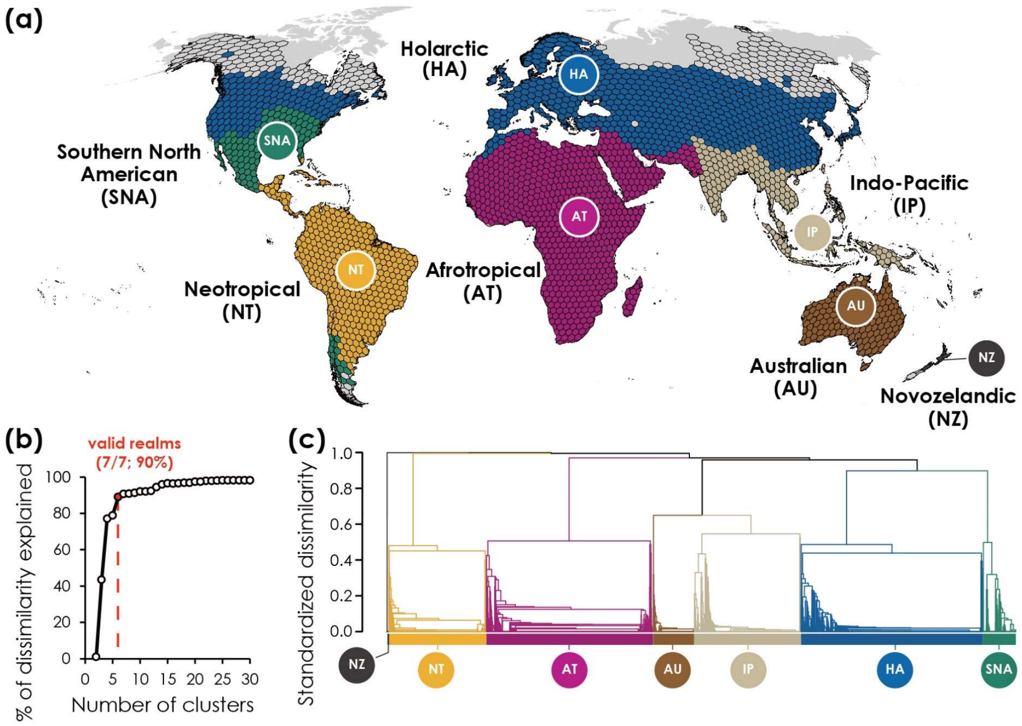

Supplementary Fig.11 Biogeographic regionalization of ant genera based on taxonomic turnover (Tβsim) by using unbiased clustering algorithm. Map of ant biogeographic units delineated (a), percentage of standardized dissimilarity explained by the number of clusters (b) and dendrogram (c) resulting from the unweighted pair-group method using arithmetic average (UPGMA) hierarchical clustering based on different geographic units. The standardized dissimilarity presented is the frequency of each pair being located in the specific number of clusters over 100 dendrogram trees. Colors used to characterize particular realms in maps and dendrograms are identical. Grey color indicates areas without sufficient data or invalid biogeographic units. Map is projected in Robinson projection system.

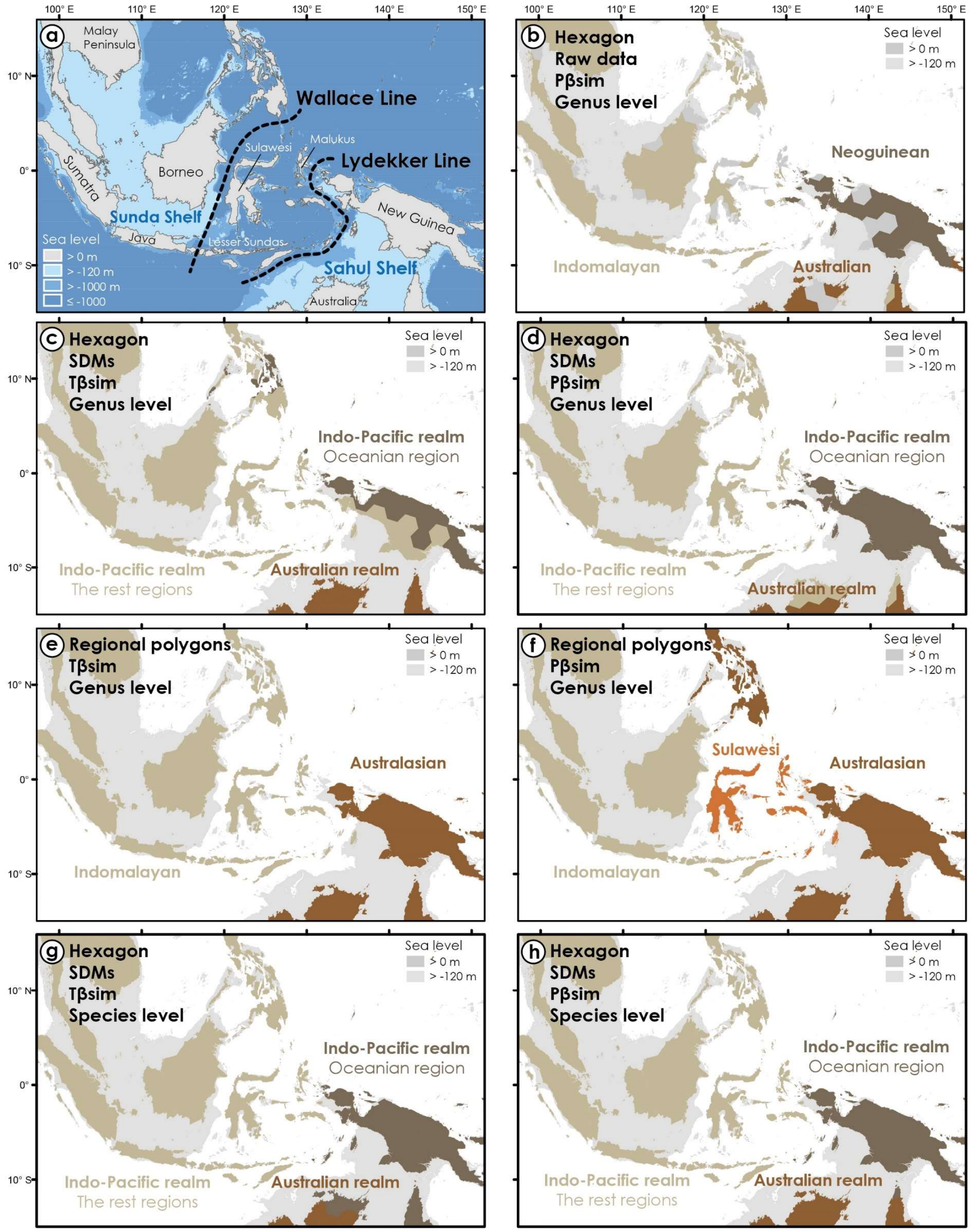

Supplementary Fig.12 Biogeographic boundaries of ants in Wallacean region based on different regionalization schemes. The geography and hypothetical biogeographic boundaries of Wallacean region (a), regionalization at genus-level based on phylogenetic turnover (Pβsim) of data from raw distribution records in 5×10^4 km^2 hexagons (b), taxonomic turnover (Tβsim) of data from species distribution modellings (SDMs) in hexagons (c), Pβsim of SDMs data in hexagons (d), Tβsim (e) and Pβsim (f) of SDMs data in regional polygons, and regionalization at genus-level based on Tβsim (g) and Pβsim (h) of SDMs data in hexagons. Regionalization based on genus-level Pβsim of raw distribution records in hexagons is not shown due to the absence of biogeographic transition in this area. Different color indicates different realms/regions. Topography information is from Ryan et al (2009).

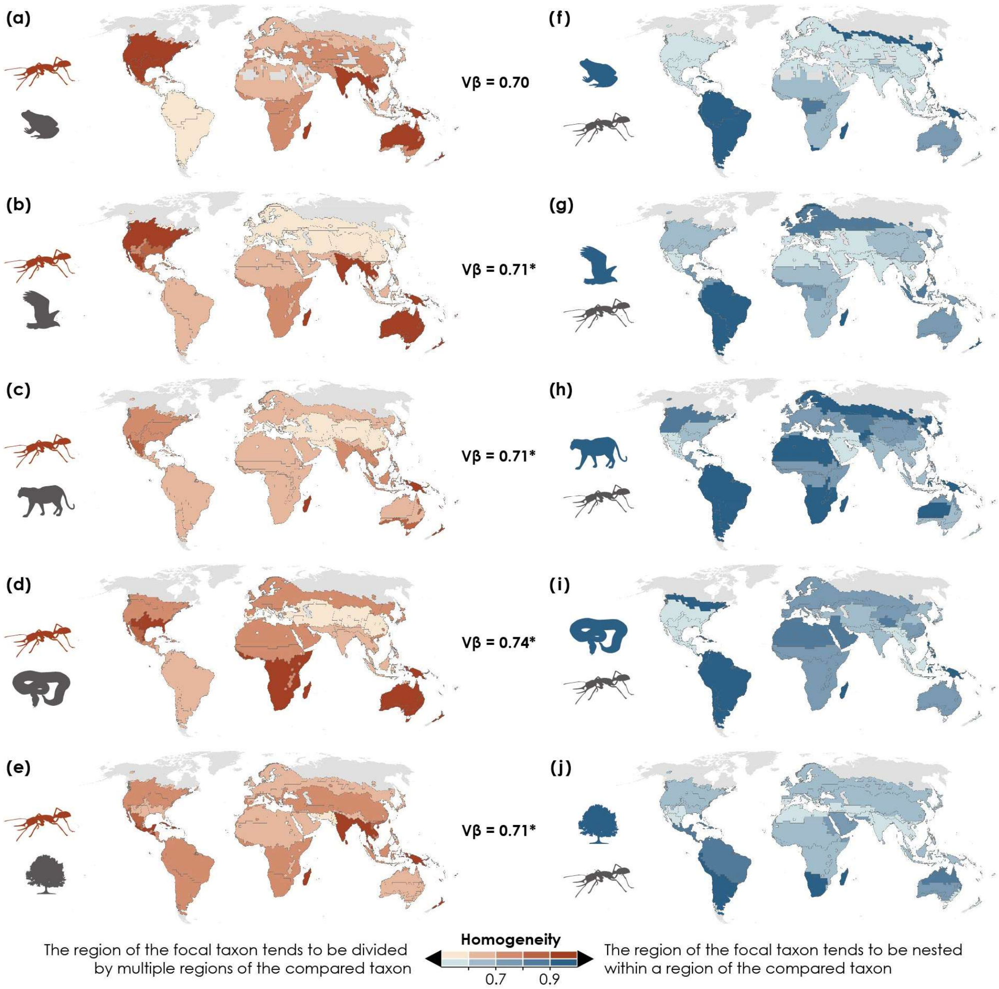

Supplementary Fig.13 The spatial association analysis shows congruence and divergence of global regionalizations between ant genera and other taxa. Colors indicate the homogeneity of biogeographic regions for ants in relation to schema for amphibians (a), birds (b), mammals (c), reptiles (d) and vascular plants (e) and the schema of these taxa in relation to the ant regionalization (f-j). Homogeneity is measured by 1 minus the normalized Shannon entropy. Darker red or blue indicates higher homogeneity. The V-measure (Vβ) is an area-weighted harmonic mean of homogeneity between two regionalization schemes, where higher values suggest stronger spatial associations between them, with * indicating that the observed biogeographic structure of ants is significantly (two-sided p value < 0.05) more similar to that of the taxon compared than would be expected by random regionalizations.

Supplementary Table 1. Evaluation of clustering algorithms. The cophenetic Pearson correlation (Cor.) and Gower distance (Gower dist.) of clustering results by using different algorithms and based on different geographic units (hexagons or regional polygons), distribution data (species distribution modellings, SDMs or raw records), taxonomic classifications (genus- or species-level) and distance metrices (Simpson index of pairwise phylogenetic dissimilarity, Pβsim or taxonomic dissimilarity, Tβsim) are shown. Abbreviations: UPGMA, unweighted pair-group method using arithmetic averages; UPGMC, unweighted pair-group method using centroids; WARD, Ward's method; SL, single lineage; CL, complete lineage; WPGMA, weighted pair-group method using arithmetic averages; WPGMC, weighted pair-group method using centroids.

Unit	Data	Pβsim			Tβsim		
		**Method**	**Cor.**	**Gow Dis.**	**Method**	**Cor.**	**Gow Dis.**
hexagon	SDMs (genus)	**average**	**0.75**	**74699.67**	**average**	**0.75**	**121450.13**
		centroid	0.74	263671.70	centroid	0.74	508107.27
		ward.D2	0.70	556978679.34	ward.D2	0.69	950086965.64
		single	0.05	877954.00	single	0.07	1606525.47
		complete	0.58	933457.30	complete	0.58	899486.10
		mcquitty	0.63	109407.77	mcquitty	0.68	148851.50
		median	0.57	346433.75	median	0.60	535028.57
	SDMs (species)	**average**	**0.78**	**65741.49**	**average**	**0.89**	**31957.70**
		centroid	0.73	787183.12	centroid	0.53	1892273.09
		ward.D2	0.70	811857026.03	ward.D2	0.78	975216921.82
		single	0.24	1659027.30	single	0.36	2399967.47
		complete	0.63	475910.21	complete	0.56	122274.37
		mcquitty	0.72	82973.45	mcquitty	0.82	50351.86
		median	0.60	763511.44	median	0.36	1741913.47
	Records (genus)	**average**	**0.68**	**41080.83**	**average**	**0.71**	**64919.71**
		centroid	0.66	168569.71	centroid	0.64	359409.03
		ward.D2	0.63	157801021.61	ward.D2	0.66	305776549.97
		single	0.07	465050.97	single	0.09	935811.54
		complete	0.52	466411.72	complete	0.43	378033.11
		mcquitty	0.63	64934.92	mcquitty	0.67	136714.87
		median	0.60	127202.27	median	0.51	392061.31
polygon	Records (genus)	**average**	**0.71**	**1947.65**	**average**	**0.73**	**2821.59**
		centroid	0.70	5575.75	centroid	0.72	10971.95
		ward.D2	0.70	1610177.84	ward.D2	0.68	2847013.39
		single	0.06	17937.62	single	0.15	32545.41
		complete	0.59	21768.89	complete	0.57	22789.76
		mcquitty	0.61	2527.90	mcquitty	0.68	3842.66
		median	0.57	6819.79	median	0.58	9496.54

References

Guénard, B., Weiser, M. D., Gomez, K., Narula, N., & Economo, E. P. The Global Ant Biodiversity Informatics (GABI) database: synthesizing data on the geographic distribution of ant species (Hymenoptera: Formicidae). *Myrmecol. News* 24, 83–89 (2017).

Ryan, W. B. et al. Global multi-resolution topography synthesis. *Geochemistry, Geophys. Geosystems* 10, Q03014 (2009).
