# Supplementary material for: Global biogeographic regions for ants have complex relationships with those for plants and tetrapods
Source: Nat Commun. 2024 Jul 5;15:5641. doi: 10.1038/s41467-024-49918-2 (PMC11226674; doi:10.1038/s41467-024-49918-2)
Supplement: Supplementary file 3 — Reporting Summary [file 41467_2024_49918_MOESM3_ESM.pdf]

nature portfolio

Corresponding author(s): Runxi Wang

Last updated by author(s): Jun 14, 2024

Reporting Summary

Nature Portfolio wishes to improve the reproducibility of the work that we publish. This form provides structure for consistency and transparency in reporting. For further information on Nature Portfolio policies, see our Editorial Policies and the Editorial Policy Checklist.

Statistics

For all statistical analyses, confirm that the following items are present in the figure legend, table legend, main text, or Methods section.

n/a	Confirmed	
☒	☐	The exact sample size (*n*) for each experimental group/condition, given as a discrete number and unit of measurement
☒	☐	A statement on whether measurements were taken from distinct samples or whether the same sample was measured repeatedly
☐	☒	The statistical test(s) used AND whether they are one- or two-sided<br>*Only common tests should be described solely by name; describe more complex techniques in the Methods section.*
☐	☒	A description of all covariates tested
☐	☒	A description of any assumptions or corrections, such as tests of normality and adjustment for multiple comparisons
☐	☒	A full description of the statistical parameters including central tendency (e.g. means) or other basic estimates (e.g. regression coefficient) AND variation (e.g. standard deviation) or associated estimates of uncertainty (e.g. confidence intervals)
☐	☒	For null hypothesis testing, the test statistic (e.g. *F*, *t*, *r*) with confidence intervals, effect sizes, degrees of freedom and *P* value noted<br>*Give P values as exact values whenever suitable.*
☒	☐	For Bayesian analysis, information on the choice of priors and Markov chain Monte Carlo settings
☒	☐	For hierarchical and complex designs, identification of the appropriate level for tests and full reporting of outcomes
☐	☒	Estimates of effect sizes (e.g. Cohen's *d*, Pearson's *r*), indicating how they were calculated

Our web collection on statistics for biologists contains articles on many of the points above.

Software and code

Policy information about availability of computer code

Data collection	No software was used to collect data
Data analysis	All analyses were conducted in the R programming version 4.2.1, we performed the regionalization analyses mainly using the R packages betapart 1.5.2, phyloregion 1.0.8, recluster 2.8; processed phylogenetic analyses using the R packages geiger 2.0.7 and picante 1.8.2; conducted spatial statistics using the R packages sabre 0.4.3 and SpatialPack 0.3-8196. All raw data and custom R codes are available from the figshare repository (https://doi.org/10.6084/m9.figshare.25011866).

For manuscripts utilizing custom algorithms or software that are central to the research but not yet described in published literature, software must be made available to editors and reviewers. We strongly encourage code deposition in a community repository (e.g. GitHub). See the Nature Portfolio guidelines for submitting code & software for further information.

Data

Policy information about availability of data

All manuscripts must include a data availability statement. This statement should provide the following information, where applicable:

- Accession codes, unique identifiers, or web links for publicly available datasets
- A description of any restrictions on data availability
- For clinical datasets or third party data, please ensure that the statement adheres to our policy

The ant distribution data are available from the Global Ant Biodiversity Informatics (GABI) database, and the reconstructed phylogenies are from Economo et al. [Economo, E. P., Narula, N., Friedman, N. R., Weiser, M. D., & Guénard, B. Macroecology and macroevolution of the latitudinal diversity gradient in ants. Nat.

Commun. 9, 1–8 (2018).]. Continuous range estimates for ant genera and species are available from the supplemental data section of Kass et al. (2022) at https://datadryad.org/stash/dataset/doi:10.5061/dryad.wstqjq2pp. The final dataset used for regionalization analysis can be found on Figshare (https://doi.org/10.6084/m9.figshare.25011866). The zoogeographic regions of amphibians, birds and mammals (Holt et al., 2013) were accessed from https://macroecology.ku.dk/resources/wallace/, reptiles (Falaschi et al., 2023) were accessed from https://doi.org/10.6084/m9.figshare.19844755 and the phylogeographic regionalization for vascular plants was obtained from Carta et al. (2022), available at https://github.com/spiritu-santi/Floristic-Kingdoms/tree/main/shapefiles.

Research involving human participants, their data, or biological material

Policy information about studies with human participants or human data. See also policy information about sex, gender (identity/presentation), and sexual orientation and race, ethnicity and racism.

Reporting on sex and gender	*Use the terms sex (biological attribute) and gender (shaped by social and cultural circumstances) carefully in order to avoid confusing both terms. Indicate if findings apply to only one sex or gender; describe whether sex and gender were considered in study design; whether sex and/or gender was determined based on self-reporting or assigned and methods used.*<br>*Provide in the source data disaggregated sex and gender data, where this information has been collected, and if consent has been obtained for sharing of individual-level data; provide overall numbers in this Reporting Summary. Please state if this information has not been collected.*<br>*Report sex- and gender-based analyses where performed, justify reasons for lack of sex- and gender-based analysis.*
Reporting on race, ethnicity, or other socially relevant groupings	*Please specify the socially constructed or socially relevant categorization variable(s) used in your manuscript and explain why they were used. Please note that such variables should not be used as proxies for other socially constructed/relevant variables (for example, race or ethnicity should not be used as a proxy for socioeconomic status).*<br>*Provide clear definitions of the relevant terms used, how they were provided (by the participants/respondents, the researchers, or third parties), and the method(s) used to classify people into the different categories (e.g. self-report, census or administrative data, social media data, etc.)*<br>*Please provide details about how you controlled for confounding variables in your analyses.*
Population characteristics	*Describe the covariate-relevant population characteristics of the human research participants (e.g. age, genotypic information, past and current diagnosis and treatment categories). If you filled out the behavioural & social sciences study design questions and have nothing to add here, write "See above."*
Recruitment	*Describe how participants were recruited. Outline any potential self-selection bias or other biases that may be present and how these are likely to impact results.*
Ethics oversight	*Identify the organization(s) that approved the study protocol.*

Note that full information on the approval of the study protocol must also be provided in the manuscript.

Field-specific reporting

Please select the one below that is the best fit for your research. If you are not sure, read the appropriate sections before making your selection.

☐ Life sciences ☐ Behavioural & social sciences ☒ Ecological, evolutionary & environmental sciences

For a reference copy of the document with all sections, see nature.com/documents/nr-reporting-summary-flat.pdf

Ecological, evolutionary & environmental sciences study design

All studies must disclose on these points even when the disclosure is negative.

Study description	To delineate the global biogeographic regionalization of ants and examined its similarity to regionalizations for tetrapods and vascular plants, we used the most recent geographic estimates for 345 genera and 14,324 species and subspecies, integrating their phylogenetic relationships to quantify the taxonomic and phylogenetic turnover of ant assemblages in equal-area hexagon grids covering the Earth's surface, and used spatial statistics to quantify the degree and significance of biogeographic associations among different taxonomic groups.
Research sample	We sampled all currently valid ant genera, species and subspecies on Earth but excluded non-native taxa and excluded those without phylogenetic information when calculating the phylogenetic turnover.
Sampling strategy	Does not apply
Data collection	The ant distribution data are available from the Global Ant Biodiversity Informatics (GABI) database which includes data from the literature (both English and non-English languages), specimen information (e.g., museum and personal collections) and unpublished datasets. The phylogenetic information of ants was derived from a recently reconstructed large-scale phylogeny of ants (Economo et al., 2018) which was grafted by 100 backbone trees of 262 terminal clades from the posterior and represented the phylogenetic relationships of >14,000 ant taxa with their uncertainty. The zoogeographic regions of amphibians, birds and mammals (Holt et al., 2013) were accessed from https://macroecology.ku.dk/resources/wallace/, reptiles (Falaschi et al., 2023) were accessed from https://doi.org/10.6084/m9.figshare.19844755 and the phylogeographic regionalization for vascular plants was obtained from Carta et al. (2022), available at https://github.com/spiritu-santi/Floristic-Kingdoms/tree/main/shapefiles.
Timing and spatial scale	The geographic and taxonomic information of ant genera and species were accessed and updated until 1st March 2023. And the

	spatial scale is the terrestrial area of Earth except Antarctica.
Data exclusions	We excluded non-native taxa and those with invalid geographic information in the dataset; excluded taxa without phylogenetic information when calculating the phylogenetic turnover; and exclude hexagons with fewer than 5 taxa assigned as present to avoid potential distortion due to small sample sizes in dissimilarity analyses.
Reproducibility	All analyses are performed within the R programming environment, and the code and the data for all analyses is deposited in the figshare repository.
Randomization	Does not apply
Blinding	Does not apply

Did the study involve field work? ☐ Yes ☒ No

Reporting for specific materials, systems and methods

We require information from authors about some types of materials, experimental systems and methods used in many studies. Here, indicate whether each material, system or method listed is relevant to your study. If you are not sure if a list item applies to your research, read the appropriate section before selecting a response.

Materials & experimental systems

n/a	Involved in the study
☒	☐ Antibodies
☒	☐ Eukaryotic cell lines
☒	☐ Palaeontology and archaeology
☒	☐ Animals and other organisms
☒	☐ Clinical data
☒	☐ Dual use research of concern
☒	☐ Plants

Methods

n/a	Involved in the study
☒	☐ ChIP-seq
☒	☐ Flow cytometry
☒	☐ MRI-based neuroimaging

Plants

Seed stocks	*Report on the source of all seed stocks or other plant material used. If applicable, state the seed stock centre and catalogue number. If plant specimens were collected from the field, describe the collection location, date and sampling procedures.*
Novel plant genotypes	*Describe the methods by which all novel plant genotypes were produced. This includes those generated by transgenic approaches, gene editing, chemical/radiation-based mutagenesis and hybridization. For transgenic lines, describe the transformation method, the number of independent lines analyzed and the generation upon which experiments were performed. For gene-edited lines, describe the editor used, the endogenous sequence targeted for editing, the targeting guide RNA sequence (if applicable) and how the editor was applied.*
Authentication	*Describe any authentication procedures for each seed stock used or novel genotype generated. Describe any experiments used to assess the effect of a mutation and, where applicable, how potential secondary effects (e.g. second site T-DNA insertions, mosiacism, off-target gene editing) were examined.*
